# Hospital Furniture

**Published:** 1893-06-24

**Authors:** 


### HOSPITAL FURNITURE.

We have received a new catalogue from Messrs. Atkinson and Co., Westminster Bridge Road, which contains drawings of almost every kind of furniture suitable for institution use, from bedsteads and mattresses to medicine cupboards. This firm devotes much attention to the fitting and furnishing of hospitals, asylums, &c., being contractors to many of our large London institutions, and we cordially recommend the aforesaid catalogue to the attention of hospital authorities. We noticed particularly excellent designs for ward tables and wash-stands.

